# Pressing Crowd Noise Impairs the Ability of Anxious Basketball Referees to Discriminate Fouls

**DOI:** 10.3389/fpsyg.2019.02380

**Published:** 2019-10-21

**Authors:** Fabrizio Sors, David Tomé Lourido, Vittoria Parisi, Ilaria Santoro, Alessandra Galmonte, Tiziano Agostini, Mauro Murgia

**Affiliations:** ^1^Department of Medicine, Surgery and Health Sciences, University of Trieste, Trieste, Italy; ^2^Department of Life Sciences, University of Trieste, Trieste, Italy; ^3^Department of Medical Area, University of Udine, Udine, Italy; ^4^Department of Psycho-Socio-Educational Analysis and Intervention, University of Vigo, Vigo, Spain

**Keywords:** referees, basketball, crowd noise, anxiety, fouls, sport, sound

## Abstract

The decision-making processes of referees in sports are affected by many factors, including the pressure of spectators. While the home/visitor bias has been previously investigated, the role of crowd noise has been less studied. In the present study, we investigated how the crowd noise (calm vs. pressing) influence the decisions of basketball referees, when examining videos of potential fouls. In doing so, we also considered the level of competitive anxiety of referees (low vs. high anxiety), as factor potentially interacting with the pressure exerted by the spectators. A 2 × 2 ANOVA (Crowd noise x Anxiety) revealed a significant interaction [*F*_(1,28)_ = 7.33; *p* < 0.05; $\eta_{\text{p}}^{2}$ = 0.21; power = 0.74], with the highly anxious referees showing poorer performances in the pressing crowd condition [*t*_(14)_ = 2.24; *p* < 0.05; *d* = 0.64]. The results indicate that the crowd noise does not seem to affect the referees' decisions, unless we consider the anxiety. The present findings suggest that the decisions of referees with high anxiety might be more easily influenced by external factors like crowd noise. Based on these results, referees' federations should consider the possibility to develop training protocols dedicated to highly anxious referees, to avoid their decisions from being biased by spectators' pressure.

## Introduction

Within the context of sport psychology research, the different elements that can influence the decisions of a judge or referee have been widely investigated (for a recent review, see Aragao-Pina et al., 2018). In fact, most of the studies that evaluate judgments or identification tasks in sports are related to decisions made by the referees (Plessner and Haar, 2006).

The process of decision-making in referees can be considered as a sequence of social information processing, since the referee is in constant interaction with the athletes and the coaches, and s/he can also be influenced by the people attending the competition, that is, the supporters. The effects of various individual and social phenomena on referees' performance have been studied, namely: the anxiety they experience when performing in competitions (Johansen and Haugen, 2013); their sports and academic training (MacMahon et al., 2007); the coping of stressful situations (Wolfson and Neave, 2007; Page and Page, 2010); the verbalizations of the athletes (Lex et al., 2015) and of the coaches (Souchon et al., 2013); the nationality of the athletes (Dawson and Dobson, 2010; Pope and Pope, 2015); the attentional biases (Pazzona et al., 2018); the order of events that take place during the competition (Plessner and Betsch, 2001; Unkelbach and Memmert, 2008; Buraimo et al., 2010); the result during matches (Lago-Peñas and Gómez-López, 2016); the condition of playing at home (Pollard, 1986; Boyko et al., 2007; Dawson et al., 2007); the importance of the supporters (Garicano et al., 2005). Altogether, the results of these studies indicate that the task of referees is quite complex and can be affected by various phenomena; among the factors constituting the complexity of refereeing, the psychological factors play an important role and can affect referee's performances to a significant extent.

Regarding the supporters, most studies have focused on the influence that various factors exert on arbitration decisions, such as the number of spectators (Downward and Jones, 2007; Pettersson-Lidbom and Priks, 2010; Picazo-Tadeo et al., 2016), the composition of the crowd, depending on whether they are local or visitors (Dohmen, 2008; Ponzo and Scoppa, 2014), the distance between the field and the stand (Scoppa, 2008; Buraimo et al., 2012), and the noise produced by the spectators (Unkelbach and Memmert, 2010). Although the relevant effect of noise on arbitration decisions was already highlighted by Greer (1983), few studies have evaluated this phenomenon experimentally (Nevill et al., 2002, 2017; Balmer et al., 2007; Unkelbach and Memmert, 2010; Myers et al., 2012).

Balmer et al. (2007) investigated the association between the bias of favoring local football teams by referees with an increase in anxiety and activation in the presence (vs. absence) of crowd noise. The results of the research indicated that, when the crowd noise was present, a bias in favor of the local teams emerged, as well as significant relationships between this bias and the increase of cognitive anxiety and perception of mental effort. The authors suggested that crowd noise is associated with an increase of anxiety and a perception of mental effort, which leads the referees facing this anxiety to take more popular decisions in favor of the local team. Thus, it seems that the referees face a double commitment: on the one hand, to be impartial due to their institutional position; on the other hand, to please the crowd (Sutter and Kocher, 2004). According to Balmer et al. (2007), as the crowd has a more immediate influence, there is a bias in favor of the team playing at home. Sometimes, arbitral inconsistencies can be explained by an avoidance coping strategy. Specifically, when facing difficult situations, referees simply avoid making unpopular decisions by ordering players to continue playing (Nevill et al., 2017).

The explanations related to favoring one team or another are marked by a strong motivational character, which would also allow the opposite outcome. Indeed, referees could show reactance to local supporters because they feel harassed (Unkelbach and Memmert, 2010). For this reason, other types of explanatory theories of the crowd noise influence on arbitration decisions have also been proposed, ranging from Bayesian decision-making models that consider some of the previously mentioned indicators (e.g., playing at home, composition and size of the crowd, number of penalties assigned; Constantinou et al., 2014), to the theory of social influence (Di Corrado et al., 2011). According to the latter theory, referees are governed by the traditional models of conformity with the majority and obedience proposed by Asch (1951) and Milgram (1963). However, this interpretation is not sufficient, according to explanations based on cognitive social theory (Plessner and Haar, 2006; Plessner et al., 2009). Considering the cognitive social theory, social knowledge and the cognitive processing of information are involved when individuals construct their subjective reality (Di Corrado et al., 2011), which in the case of the present study corresponds to the refereeing decisions. These decisions would be similar to those made in a categorization task, where the existence or not of an infraction must be determined.

Within the framework of cognitive research with sports referees, cue learning emerges (Plessner et al., 2009; Unkelbach and Memmert, 2010). This learning is due to the nature of the game in sports such as basketball or soccer, where it is continuous and dynamic, so referees often need to make quick decisions with limited information (Morris and Lewis, 2010). The latter authors point out that referees use temporary contiguity cues as additional sources of information, which are used for the decision-making process. When judging actions such as fouls, not all information is accessible at the same time (for example, limited visual information due to perspective), so concurrent cues such as noise from the crowd can be used (Plessner et al., 2009). Referees can exploit that cue because they learn the correlation between crowd noise and specific criteria in a given situation (Unkelbach and Memmert, 2010). Therefore, the prior experience of the referees with the environmental pressure will influence the signaling or not of an infraction, allowing them to improve the decision making through specific training (Webb et al., 2018). In addition, the signaling or not of an infraction may be conditioned by the anxiety levels of the referees (Balmer et al., 2007).

In the present study, rather than analyzing the effect of the home crowd or the compliance with an arbitration decision based on crowd noise, the aim was to investigate the influence of a calm vs. pressing crowd noise on the ability of basketball referees to discriminate contact situations as regular or not; in particular, in doing so the competitive anxiety characterizing the referees themselves was also considered. On the basis of the aforementioned studies, and considering that participants were not informed about the condition of “local” and “visiting” teams, the research hypotheses were the following: (1) independently from anxiety levels, the ability to correctly discriminate between fouls and non-fouls would be lower in the pressing crowd condition than in the calm crowd condition; (2) considering anxiety levels, the discrimination ability of referees with high anxiety would be more impaired than that of referees with low anxiety in the pressing crowd condition, while no differences would emerge in the calm crowd condition.

## Materials and Methods

### Participants

Thirty male basketball referees voluntarily participated in the experiment. Their age ranged between 18 and 60 years (M = 27.8; SD = 11.8). They were all active at the time of experimentation, serving as referees from youth leagues to the Italian national second division; their experience ranged between 3 and 35 years (M = 10.3; SD = 9). Participants were divided in two equally numerous groups—low vs. high anxiety (*N* = 15 each)—on the basis of the sample's anxiety median score; the two resulting groups were comparable in terms of age (low anxiety: M = 29.3 years; SD = 12.3; high anxiety: M = 26.3 years; SD = 11.1) and experience (low anxiety: M = 11 years; SD = 9.3; high anxiety: M = 9.7 years; SD = 8.9).

All participants had normal or corrected-to-normal vision, and reported no hearing disturbances. Written informed consent was obtained from each participant prior to the beginning of the experiment, in accordance with the Declaration of Helsinki. The experimental protocol was approved by the Ethics Committee of the University of Trieste, and the experiment was carried out in accordance with its recommendations.

### Materials

#### Instruments

The software iMovie and Adobe Audition 3.0 were used to edit video and audio materials, respectively. A laptop computer Dell Inspiron 5559 with a 15.6” LED display was used to test participants. Sennheiser HD 205 II circumaural headphones were used to convey the auditory stimuli. The experiment was programmed with the software E-prime Professional 2.0. The statistical software SPSS 25.0 for Windows was used for data analysis.

#### Stimuli

The stimuli used for the present experiment were videos of basketball actions containing contact situations; the original audio was removed and replaced with that of a calm crowd and that of a pressing crowd.

The videos suitable for the experiment were selected from an archive of videos used by referee instructors during technical meetings. In particular, three instructors were requested to independently view about 80 of such videos, and to judge whether the specific contact situations were to be considered as fouls or non-fouls; this independent evaluation yielded 17 unanimous fouls and 15 unanimous non-fouls. Out of these, the three instructors were requested to collectively select 10 fouls and 10 non-fouls, by discarding the most evident cases for each category, to avoid the experimental task from being too easy. Then, these 20 videos were edited so that all of them lasted 5 s, being temporally occluded immediately after the contact to be judged.

**Figure 1:**
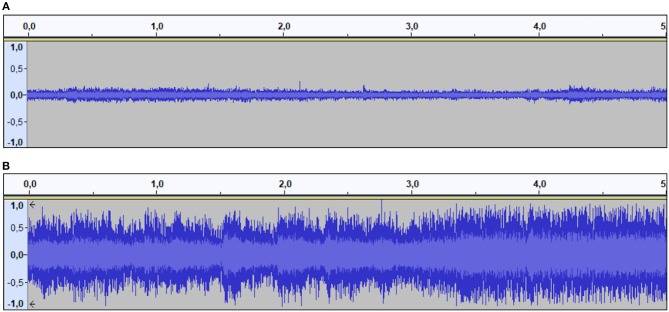
The waveforms of the audio tracks characterizing the calm crowd condition **(A)** and the pressing crowd condition **(B)**.

As concerns the audio, the same three instructors as above were asked to evaluate some tracks extrapolated from videos of basketball matches found online. In Figure 1 there are the waveforms of the two selected tracks. Beyond the quantitative differences that can be seen in the figure, the two tracks were also qualitatively different. Indeed, the calm crowd track consisted of a continuous background noise of some voices, above which the ball bounces and the players' footsteps were clearly audible. Instead, the pressing crowd track started with a drum noise lasting about 3 s, and then, after few audible footsteps, the crowd started to scream (right after the contacts to be judged by participants). These two tracks were used to replace the original audio of each of the 20 selected videos; as a consequence, the final set of stimuli consisted of 40 videos: 10 fouls with the calm crowd; 10 fouls with the pressing crowd; 10 non-fouls with the calm crowd; 10 non-fouls with the pressing crowd. An example of each can be found in the Supplementary Material.

#### Questionnaire

To measure participants' competitive anxiety, the Sport Anxiety Scale (SAS; Smith et al., 1990) was used. This questionnaire consists of 21 items in the form of statements describing thoughts and feelings that can be commonly experienced before and during sport competitions. With respect to the original version, in the instructions the three occurrences of the term “athletes” were replaced with the term “referees.” The answer to each item is to be given on a four-point Likert scale, ranging from “not at all” (1) to “very much so” (4).

The items compose two subscales, i.e., somatic anxiety and cognitive anxiety; the latter can be further divided in two sub-subscales, i.e., worry and concentration disruption. A total competitive anxiety score can also be calculated by adding all the items together. In the present study, we used this total competitive anxiety score, in order to create two groups of participants (low vs. high anxiety).

### Procedure and Task

Participants were tested individually in a quiet room. Upon their arrival, they were asked to seat in front of a laptop computer and to wear the headphones; then, the experimenter launched the experimental session. Participants were instructed that their task was to judge whether each contact was a foul or a non-foul, by pressing one of two keys on the laptop keyboard, namely “A” or “L”^1^; in the instructions, no reference was made to the crowd noise.

The experimental session consisted of two blocks, each composed of 20 trials, for a total of 40 trials (corresponding to the 40 stimuli). In each block there were five fouls with the calm crowd, five fouls with the pressing crowd, five non-fouls with the calm crowd, and five non-fouls with the pressing crowd. The stimuli were presented in a randomized order within each block; moreover, within each block the same action was never presented twice (e.g., foul “A” with the calm crowd was presented in the first block, while the same foul “A” but with the pressing crowd was presented in the second block). After each response, there was an interval of 1 s before the beginning of the subsequent stimulus; instead, the interval between the two blocks was of 5 min, to provide participants with an appropriate rest.

Once the experimental session was completed, participants were administered the SAS. The choice to administer it at the end of the protocol rather than at its beginning was made to avoid a possible “triggering” of anxiety-related thoughts/feelings before performing the task, which could have led (some) participants to explicitly focus on the crowd noise as a potential anxiogenic stimulus.

### Statistical Analyses

First of all, the *d'* scores for each participant in each condition—calm crowd and pressing crowd—were calculated, as measures of response accuracy. Subsequently, the competitive anxiety score for each participant was calculated. Then, participants were divided in two equally numerous groups—low vs. high anxiety (*N* = 15 each)—on the basis of the sample's median score. To test the research hypotheses, a 2 × 2 mixed ANOVA was conducted, with crowd noise (calm vs. pressing) as within-subjects variable, and anxiety (low vs. high) as between-subjects variable. Moreover, we conducted planned comparisons between the two groups, in each of the two crowd noise conditions, as well as within each group in the two crowd noise conditions, by means of a set of independent samples *t*-tests, and paired samples *t*-tests, respectively. For the ANOVA, the sphericity assumption was evaluated using the Mauchly test; Greenhouse-Geisser correction for degrees of freedom was applied in case of non-sphericity. The effect sizes were calculated using the partial eta square ($\eta_{\text{p}}^{2}$) (Lakens, 2013), with 0.01, 0.06, and 0.14 considered small, medium, and large effects, respectively. In the case of *t*-tests, effect sizes were calculated using the Cohen's d (Cohen, 1988), for which 0.20, 0.50, and 0.80 are considered small, medium, and large effects, respectively.

## Results

**Figure 2:**
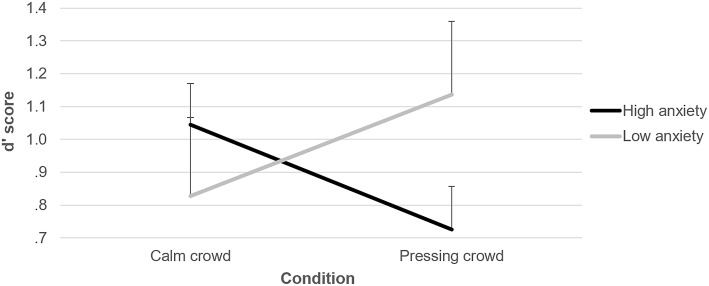
The *d'* scores of referees with low and high anxiety in the calm and pressing crowd conditions. Error bars show the standard error of the mean.

The mixed ANOVA did not reveal a significant main effect either for crowd noise [*F*(1, 28) = 0.01; *p* = 0.97] or for anxiety [*F*(1, 28) = 0.16; *p* = 0.69]; however, a significant interaction emerged [*F*(1, 28) = 7.33; *p* < 0.05; $\eta_{\text{p}}^{2}$ = 0.21; power = 0.74] (Figure 2). The set of independent samples *t*-tests revealed no difference between the two groups in the calm crowd condition [*t*(28) = 0.81; *p* = 0.43; low anxiety: M = 0.83, SD = 0.93; high anxiety: M = 1.04, SD = 0.49]; instead, in the pressing crowd condition a marginally significant difference between the two groups emerged [*t*(28) = 1.59; *p* = 0.063; *d* = 0.58; low anxiety: M = 1.14, SD = 0.86; high anxiety: M = 0.73, SD = 0.51]. The set of paired samples *t*-tests highlighted a significant difference in the two conditions for referees with high anxiety, showing lower discrimination ability in the pressing crowd condition [*t*(14) = 2.24; *p* < 0.05; *d* = 0.64]; instead, no difference emerged for referees with low anxiety between the calm and pressing crowd conditions [*t*(14) = 1.69; *p* = 0.11].

## Discussion

The aim of the present study was to investigate the influence of crowd noise on the ability of basketball referees to discriminate contact situations as regular or not, also on the basis of their competitive anxiety. Unlike previous studies on similar issues, here the manipulation did not simply consist in the presence vs. absence of crowd noise (e.g., Balmer et al., 2007) or in the variation of its volume (e.g., Unkelbach and Memmert, 2010); instead, to be more realistic, two different tracks were used—one of a calm crowd and one of a pressing crowd—with both quantitative and, most importantly, qualitative differences between them. Moreover, the condition of “local” and “visiting” teams was intentionally not specified to participants, as the phenomenon under investigation was different—more general—than the *home bias* itself. With these premises, and on the basis of previous research mentioned in the introduction, two hypotheses were tested: (1) independently from anxiety levels, the ability to correctly discriminate between fouls and non-fouls would be lower in the pressing crowd condition than in the calm crowd condition; (2) considering anxiety levels, the discrimination ability of referees with high anxiety would be more impaired than that of referees with low anxiety in the pressing crowd condition, while no differences would emerge in the calm crowd condition.

The results of the experiment seem not to support the first hypothesis, as no difference in fouls discrimination ability emerged between the calm and pressing conditions. The explanation of this outcome can be easily found in the significant interaction between crowd noise and anxiety levels; thus, the overall apparent null effect of crowd noise was actually due to its different impact on participants on the basis of their competitive anxiety. Such an interaction, together with the decreased discrimination ability of referees with high anxiety in the pressing crowd condition, as well as the absence of difference between the two groups in the calm crowd condition, seems to support the second hypothesis. A reasonable explanation of this outcome refers to cue learning, with highly anxious referees being more prone than lowly anxious ones to rely on a salient concurrent cue (like pressing crowd noise), when judging contact situations. However, such a cue can often be misleading or at least ambiguous, thus leading to a poorer performance. Another possible explanation refers to the phenomenon of *choking under pressure*, with more anxious people/athletes being more prone to experience performance decrements in pressing situations (e.g., Otten, 2009). It is also possible to hypothesize a combination of these two explanations: an anxious referee could be performing poorly during a pressing competition, and becoming aware of this s/he could try to improve her/his performance by basing her/his decisions also on crowd reactions, which in most cases would further negatively affect the performance itself. Further studies are needed to deepen the understanding of the mechanisms underpinning the processes that led to the outcomes observed in the present experiment.

An interesting result of the present experiment is the marginally significant difference between referees with high and low anxiety in the pressing crowd condition. Looking at Figure 2, it appears quite evident that the trend of referees with low anxiety is opposite to that of referees with high anxiety (as confirmed by the significant interaction). However, for the low anxiety group the comparison between the two crowd noise conditions revealed no significant difference; this is reasonably due to the higher variability with respect to the high anxiety group. Anyway, it appears that the discrimination ability of at least some referees with low anxiety was higher in the pressing crowd condition than in the calm crowd condition; this observation is in line with the phenomenon of *clutch* performances, that is, a performance increase in pressing situations (Otten, 2009), and deserves further investigation to be clarified.

In the present experiment, participants were divided in two groups—referees with low and high anxiety—on the basis of the sample's median score. In future studies investigating similar issues, it would be interesting to use questionnaires/scales with cut-off values, so that participants can be divided in two (or more) groups on more grounded bases. However, existing questionnaires with these characteristics might not suit a specific population like referees, so an alternative solution to make the results more generalizable can be that of expanding the sample size. Another aspect to consider in order to foster the generalizability of the results of future studies is the use of the latest advances in technology, such as virtual reality and/or 3D displays (also for auditory stimuli), as they allow for more ecological experimental settings (Craig, 2013).

Interestingly, the present study significantly adds not only to the literature concerning referees' decision making, but also to the line of research that in recent years is revealing the relevant role of ecological sounds in sports (for a recent review, see Schaffert et al., 2019). Indeed, previous studies highlighted that ecological auditory information can be used by athletes to perceive different features of sport gestures (Murgia et al., 2012; Kennel et al., 2014; Sors et al., 2017), to predict the outcome of opponents' actions (Allerdissen et al., 2017; Camponogara et al., 2017; Cañal-Bruland et al., 2018; Sors et al., 2018), and to improve performances (Agostini et al., 2004; Sors et al., 2015; Pizzera et al., 2017). The present study indicates that the ecological auditory information is somehow important not only for athletes, but also for referees, as it provides them with an additional source of information. Independently from the fact that this information can be useful or misleading, our findings indicate that crowd noise can affect the referees' decisional processes and, consequently, their behavior.

From an applied perspective, the results of the present experiment highlight the importance of referees' competitive anxiety levels in relation with crowd noise. Indeed, while the ability of referees with low anxiety to discriminate fouls does not significantly change with a calm crowd or a pressing one, for referees with high anxiety such ability significantly decreases with a pressing crowd noise, thus determining poorer refereeing performances. Referees' federations should be aware of this phenomenon, as it may influence competitions' results to a significant extent. This does not mean that highly anxious referees should be relegated only to less important matches (where smaller/calmer crowds are expected); rather, federations should consider the possibility to develop dedicated training protocols and/or to include in their staff the figure of the sport psychologist, who could provide specific interventions.

To sum up, the present study highlighted that pressing crowd noise impairs the ability of basketball referees with high anxiety to discriminate fouls. Further research is needed to better understand why this is the case (e.g., which factors cause this bias), as well as whether it replicates also in other sports. Findings should raise the awareness of referees' federations on this and related phenomena, in order to adopt appropriate intervention strategies to avoid competitions' results from being biased by them.

## Data Availability Statement

The raw data supporting the conclusions of this manuscript will be made available by the authors, without undue reservation, to any qualified researcher.

## Ethics Statement

The studies involving human participants were reviewed and approved by Ethics Committee of the University of Trieste. The patients/participants provided their written informed consent to participate in this study.

## Author Contributions

FS, IS, and MM conceived the idea. FS, DT, VP, IS, AG, TA, and MM designed the study. FS and VP prepared the stimuli. FS, IS, and AG programmed the experiment. FS, DT, and VP collected the data. FS, MM, and TA analyzed the data. FS, DT and MM wrote the first draft of the manuscript. VP, IS, AG, and TA revised the manuscript.

### Conflict of Interest

The authors declare that the research was conducted in the absence of any commercial or financial relationships that could be construed as a potential conflict of interest.

## Footnotes

^1^The correspondence between the keys and the answer associated to them was inverted between the two blocks, in order to keep under control any potential effect of the dominant hand.

## Supplementary Material

Video S1 Example of foul with calm crowd noise.

Video S2 Example of foul with pressing crowd noise.

Video S3 Example of non-foul with calm crowd noise.

Video S4 Example of non-foul with pressing crowd noise.
